# DICER1-associated central nervous system sarcoma with neural lineage differentiation: a case report

**DOI:** 10.1186/s13000-022-01252-1

**Published:** 2022-09-24

**Authors:** Kun Yao, Zejun Duan, Jing Feng, Changxiang Yan, Xueling Qi

**Affiliations:** 1 Department of Pathology, Sanbo Brain Hospital, Capital Medical University, No.50 Xiangshan Yikesong Road, Haidian District, 100093 Beijing, China; 2 Department of Neurosurgery, Sanbo Brain Hospital, Capital Medical University, 100093 Beijing, China

**Keywords:** DICER1-associated central nervous system sarcoma, Neurogenic differentiation, Whole-exome sequencing, Case report, Radiotherapy

## Abstract

**Background:**

*DICER1*-associated central nervous system sarcoma (DCS) without evidence of other cancer-related syndromes is rare. Though the morphology of DCS was highly variable, the immunophenotype was predominant myogenic phenotype. Other lineage markers were consistently negative.

**Case presentation:**

We report a case of DCS with neurogenic differentiation proved by immunohistochemical staining and whole-exome sequencing (WES). An 8-year-old female patient presented with 8-day history of headache, nausea and vomiting. Magnetic resonance imaging (MRI) revealed a heterogeneous mass in the left parietal lobe. The patient underwent the craniotomy via left parietal approach to resect the tumor completely. Histologically, the tumor predominately showed fibrosarcoma-like spindle cells with obvious cytoplasmic eosinophilic globules. Immunohistochemically, the tumor stained positively for DICER1, Desmin, and several neurogenic markers. *DICER1* somatic hotspot mutation was confirmed by WES, as well as *TP53* and *RAF1* mutations which were commonly found in DCS, and other sarcoma-associated genes including *AR*, *AXL* and *ETV5* mutations. Subsequently, the result of Gene Ontology (GO) analysis showed that the mutated genes in this case were involved in neuron development. All of these findings indicated the diagnosis of DCS with neurogenic differentiation. Postoperatively, the patient received high-dose radiotherapy (60 Gy) and chemotherapy. There was no MRI evidence of tumor recurrence at the 21-month postoperative follow-up.

**Conclusions:**

This unusual DCS case with neuronal differentiation is an important addition to the immuno-phenotypic spectrum of DCS. Although the prognosis for DCS is poor, gross tumor resection with high dose radiotherapy and chemotherapy may assist in prolonging survival.

**Supplementary Information:**

The online version contains supplementary material available at 10.1186/s13000-022-01252-1.

## Background

Intracranial sarcomas are uncommon tumors with poor prognoses and mainly occur in adult patients [1]. They rarely affect children, which are often associated with syndromic disorders [2–4]. DICER1 syndrome is one of rare syndromic disorders with intracranial sarcomas, which always exhibits germline *DICER1* mutations [5, 6]. Few studies reported rare cases of pediatric intracranial sarcoma involving somatic *DICER1*-mutants and without evidence of other peripheral tumors. Koelsche et al. reported a group of predominantly pediatric intracranial spindle cell sarcomas to be called “spindle cell sarcoma with rhabdomyosarcoma-like features, *DICER1* mutant (SCS-RMS like-DICER1).” In this study, the somatic *DICER1* mutation was detected in three of five cases [7]. In another study, Kamihara et al. reported six cases of “DICER1-associated central nervous system sarcoma (DCS)”, in which three of six cases harbored somatic *DICER1* mutations instead of germline *DICER1* mutations [8]. Moreover, most of the reported cases showed highly variable morphology, but they were consistent with the positive of myogenic markers, while other lineage markers were consistently negative, such as GFAP, OLIG2, SOX10, SOX2, CD34, S100, EMA, and cytokeratin [5–10]. To the best of our knowledge, there is no report of DCS with the expression of neurogenic markers. Herein, we describe a rare DCS case with the somatic *DICER1* mutation and neural lineage differentiation, including clinical, imaging, pathological, molecular profiles and treatment.

## Case presentation

An 8-year-old female presented to the neurosurgery department with intermittent headache, nausea and vomiting for 8 days. Neurological examination revealed no abnormalities. She had no familial cancer or extracranial tumor history.

### Neuroimaging

**Fig. 1:**
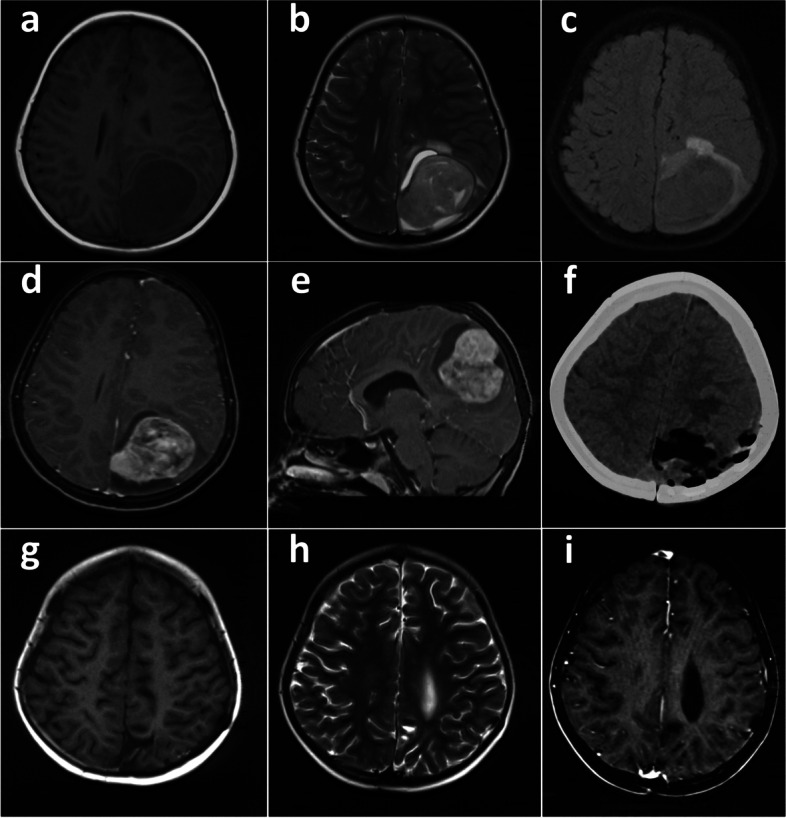
Preoperative and postoperative neuroimages of this DCS patient. Preoperative head MRI shows there is a heterogeneous intracranial mass. Axial T1-weighted MRI shows hypo-intense lesion in the left parietal lobe (**a**). Axial T2-weighted MRI shows inhomogeneous hyper-intense (**b**). Axial FLAIR image shows iso-intense (**c**). Axial and sagittal post-contrast T1-weighted MRI shows enhancement of the mass and the adjacent dura (**d** and **e**). Postoperative CT presents completely tumor resection (on the day after surgery) by axial CT (**f**). Postoperative MRI shows no tumor recurrence (19 months after surgery) which confirms on T1-weighted (**g**), T2-weighted (**h**), and axial coronal post-contrast T1-weighted (**i**)

The brain MRI revealed a heterogeneous mass in left parietal lobe which was predominantly hypointense on T1 (Fig. 1a), inhomogeneous hyperintense on T2 (Fig. 1b), and iso-intense on fluid attenuation inversion recovery (FLAIR) (Fig. 1c). After the administration of contrast, the mass displayed heterogeneous enhancement (Fig. 1d and e). The patient underwent a craniotomy via left parietal approach, which revealed a well-defined, soft, gray and gelatinous lesion attached to the falx cerebri (5 cm × 5 cm × 5 cm). The postoperative course was uneventful and postoperative CT scan showed that the intracranial tumor had been completely removed (Fig. 1f).

### Pathology and immunochemistry

**Fig. 2:**
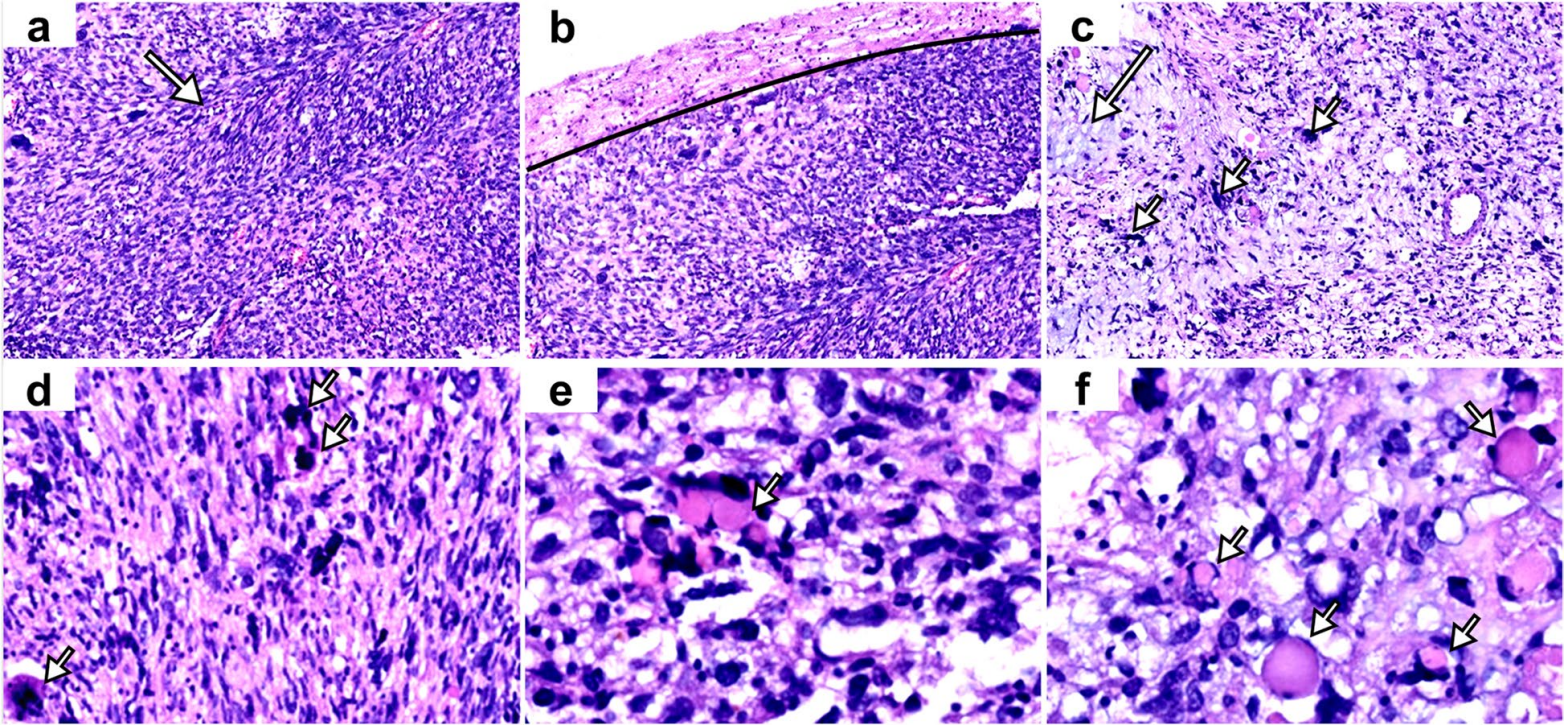
Histopathological features of the DICER1-associated central nervous system sarcoma. Histopathological features were similar with sarcomatous neoplasms. The tumor presented with spindle-shaped cells with a fascicular pattern of growth (the long arrow) (**a**). The interface of tumor and normal brain was well demarcated (the black line shows the interface) (**b**). Focal myxoid stroma matrix (the long arrow), a few multinucleated giant cells (the short arrows) (**c**), frequent mitoses (the short arrows) (**d**), and cytoplasmic eosinophilic globules (the short arrows) (**e** and **f**) were observed. Histopathological images were taken at a magnification of 100 × in a to c, 400 × in d to f

Light microscopy examination revealed sarcomatous neoplasms predominantly presented with spindle-shaped cells in a fascicular pattern (Fig. 2a). There was a clear boundary between tumor and peripheral brain tissue (Fig. 2b). In some areas, a myxoid stroma matrix was found with a few multinucleated tumor giant cells (Fig. 2c). The nuclei of the tumor cells were with brisk mitotic activity (Fig. 2d). Prominent cytoplasmic eosinophilic globules were easily identified (Fig. 2e and f).

**Fig. 3:**
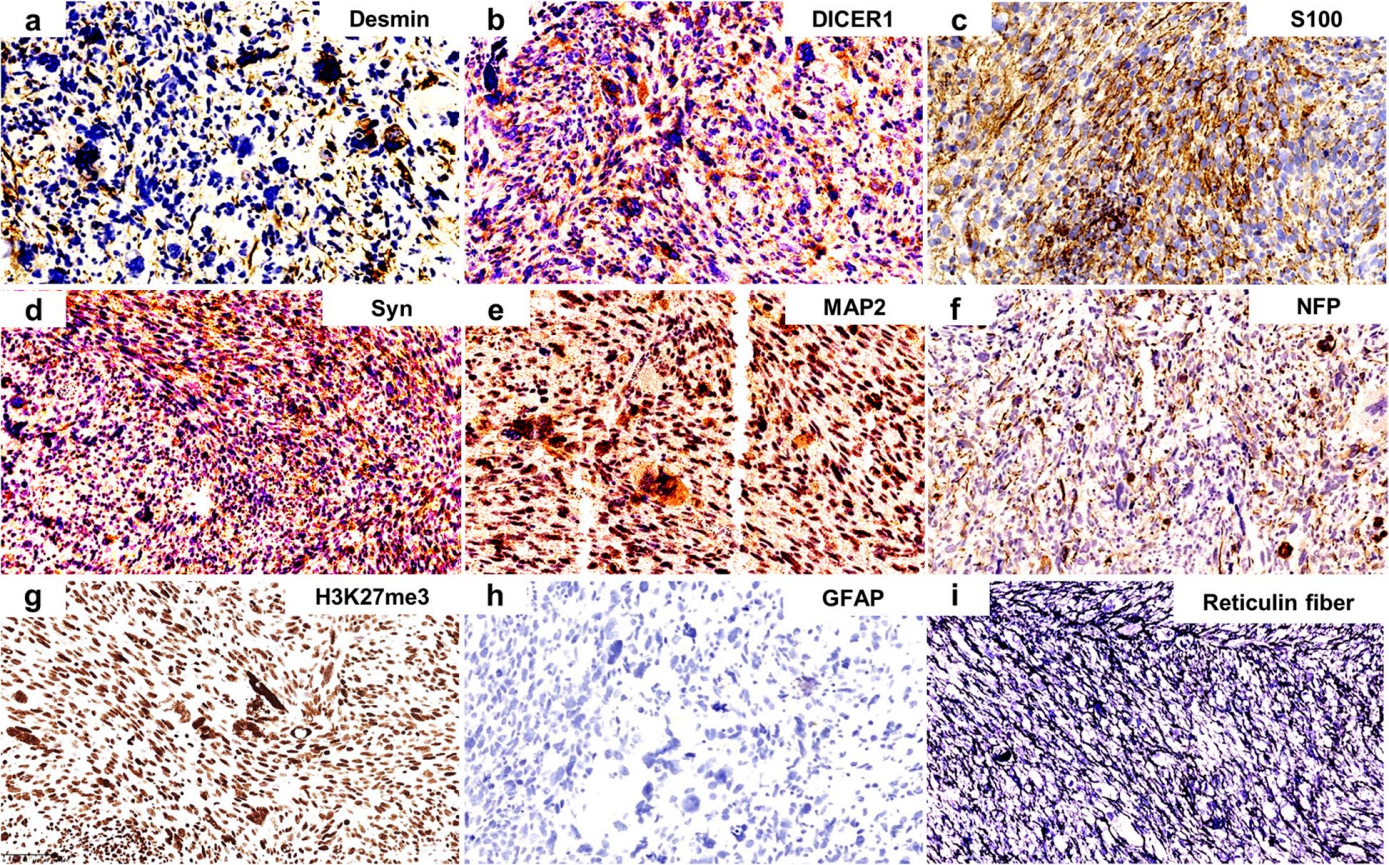
Immunohistochemical features of the DICER1-associated central nervous system sarcoma. Desmin highlighted myogenic differentiation (**a**). DICER1 protein expressed in the majority of tumor cells (**b**). S100 immunohistochemistry was positive (**c**). Strong Syn immunostaining was observed in a significant number of tumor cells (**d**). MAP2 expressed in majority of neoplastic cells (**e**). NFP immunostaining was positive in some tumor cells (**f**). The nuclear positivity of H3K27me3 staining indicated no loss of (**g**). Tumor cells were negative for GFAP (**h**). There were a large of reticulin fibres (**i**). All images were taken at a magnification of 200 ×

Immuno-histochemical staining was performed to diagnose and identify the direction of tumor differentiation by Ventana Benchmark Ultra automated stainer (Ventana, Tucson, Arizona, USA) (Fig. 3a-h, and Supplementary Table 1). Firstly, we labeled markers commonly used in the diagnosis of meningioma and non-meningothelial mesenchymal sarcoma. But the tumor cells were only stained positively for Vimentin and had focal expression of Desmin. Considering that the primary location of the tumor was intracranial, the diagnostic markers for neuroepithelial tumors were also stained. The tumor cells were stained positively for some neurogenic makers, for instance S100, Syn, MAP2, NFP, Nestin, CD56, and SOX2, which highlighted the neural lineage differentiation of tumor cells. However, the tumor cells were negative for GFAP, OLIG2, CgA, and SOX10. It also failed to provide strong diagnostic evidence. The possibility of rare tumors began to be considered. INI1 and BRG1 were retained. The DICER1 protein immunostaining was extensively positive in the tumor cells, indicating the high possibility of *DICER1* mutation. Moreover, though the frequent nuclear positivity of TLE1, loss of H3K27me3 and loss of ATRX expression in DCS has been reported [8, 11], it was not observed in this case. In addition, the P53 expression was negative. The tumor cells displayed with a large amount of thin reticulin fibers (Fig. 3i). In summary, a diagnosis of primary intracranial spindle cell sarcoma with neurogenic and myogenic differentiation, possibly associated with *DICER1* mutation, was favoured.

### Next-generation sequencing

Next, WES was performed in surgical formalin-fixed paraffin-embedded (FFPE) tumor tissues and paired normal control (peripheral blood) in order to determine whether this patient harbored somatic *DICER1* mutation and other genetic variants. In brief, WES analysis sequencing libraries were generated using Agilent SureSelect Human All Exon V6 kit (Agilent Technologies, CA, USA). DNA libraries were sequenced on Illumina Hiseq platform and 150 bp paired-end reads were generated and cleaned. The reads were mapped to the reference human genome b37 by Burrows-Wheeler Aligner (BWA) software [12]. The identification and filter of SNPs followed the workflow of GATK [13]. The parameters were set as default. The somatic mutation of *DICER1* p.E1813D (c.5439G > T, VAF: 76.47%) located within the Ribonuclease III domain was detected. The loss of heterozygosity (LOH) event in *DCIER1* was identified, which caused by deletion of the wild-type allele. And other mutations frequently mutated in DCS were detected, including *TP53* mutation (c. 560-2A > T, 81.29%) and *RAF*1 mutation (p.R191T, 40.96%) which was predicted to cause activation of the MAP kinase signaling pathway. Moreover, several sarcoma-associated gene mutations were found, including *AR* mutation (p.G473del, 11.11%), *AXL* mutation (p.T45P, 8.11%), and *ETV5* mutation (p.F11Y, 33.33%). Hence, with *DICER1* mutation accompanied by LOH and several DCS frequent mutations detected by WES, a final diagnosis of DCS was established.

**Fig. 4:**
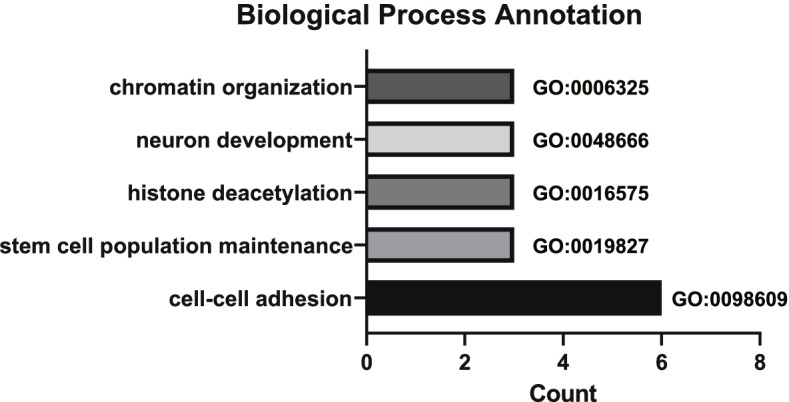
Gene Ontology (GO) annotations enriched in the somatic mutations of this DCS case. Diagrammatic sketch showed top five GO Biological Process annotations. The GO terms were sorted by *p*-value in descending order. The smaller *p*-value represents higher significance level

The result of analysis based on WES indicated that the tumor presented a high level of tumor mutational burden (TMB, 5.63 Mut/MB) as reported before [8], while showing no microsatellite instability (MSI). In addition to the mutations described above, 158 other somatic alterations were detected. To identify the potential molecular function of these variant genes, we performed Gene Ontology (GO) analysis using DAVID website [14]. The parameters were set as default. The result of GO analysis showed that mutated genes in this case were mainly involved in chromatin organization (*SATB1, ATAD2* and *CHD4*), neuron development (*TENM4, SECISBP2* and *HS6ST1*), and histone deacetylation (*SIN3B, CHD4* and *MTA2*) processes (Fig. 4). Meanwhile, RNA sequencing (RNA-seq) was performed as previously described [15], but we did not find any gene fusions.

### Post-operation

Subsequently, the patient received postoperative radiotherapy (60 Gy/30f) with the following cyclophosphamide, doxorubicin and vincristine for the first, third, fifth and seventh chemotherapy cycles. In the second, fourth, sixth and eighth cycles, ifosfamide and etoposide were used for chemotherapy. After eight cycles (median duration of 21 days among cycles) of combination chemotherapy, brain MRI revealed no evidence of tumor recurrence at the 19-month follow-up (Fig. 1g-i). At the 21-month postoperative follow-up, no clinical symptoms were observed.

## Discussion and conclusions

We here describe a case of DCS harboring somatic DICER1 hotspot mutation with neurogenic differentiation. Early reports have described that though the morphology of DCS was highly variable, spindle cell morphology was common with immunopositive myogenic markers (SMA, desmin, and myogenin) [7, 8]. Other lineage markers were consistently negative [7, 8]. In the current case, the tumor predominately showed fibrosarcoma-like spindle cells with brisk mitotic activity, including obvious cytoplasmic eosinophilic globules. These microscopic features were quite similar to previous reports [7, 8]. Unlike previous cases, this case showed the expression of neurogenic markers (S100, NFP, Syn, MAP2, CD56, Nestin, and SOX2) in addition to myogenic marker (Desmin). The GO analysis based on somatic mutations called by WES had indicated that several genes involved in neuron development were mutated, such as *TENM4, SECISBP2* and *HS6ST1*. We have shown that this case had both neural characteristics and the potential for neural development. The neurogenic differentiation was found in DCS, and this testified the statement of "highly variable heterologous differentiation of DCS" on the other side.

Except for somatic *DICER1* hotspot mutation, some other genetic alterations were detected in this patient. The *TP53* mutation and other mutations related to the MAP-kinase pathway were frequent in SCS-RMS like-DICER1 [7, 8, 10, 11]. *TP53* mutation and *RAF1* gene mutation, which were associated with MAP-kinase pathway, have been observed in the current case too. Furthermore, the patient also carried tumor-driving genes: *AR, AXL* and *ETV5* mutations. Recently, *AXL* has been proven to be a candidate for pathogenesis and therapeutic investigations in leiomyosarcoma [16] and osteosarcoma [17]. It was demonstrated that *ETV5* had oncogenic function on Ewing's sarcoma [18]. This may be of interest because *AXL* and *ETV5*, confirmed sarcoma-associated genes in leiomyosarcoma, osteosarcoma and Ewing's sarcoma growth, can be also detected in DCS cases [16–18]. Similar tumor-driving mutations in this DCS and other kinds of sarcoma suggested that there was also an inherent relationship between DCS and some other mesenchymal sarcomas.

Clinical experience with DCS is very limited. Some DCS cases without germline *DICER1* variants and family history of *DICER1*-related tumors were presumed to represent sporadic disease [8]. It is worth noting that a few DCS cases without germline *DICER1* variants may present de novo neoplasm in other organs besides intracranial sarcoma as time goes on [7]. In our case, however, no other diseases occurred during the 21 months of follow-up after surgery. Generally, even if the DCS patients without *DICER1* germline mutation did not have any other diseases at the time of DCS, it is still required to pay close attention to their general and systemic examination in postoperative follow-up.

Sporadic reports of long-term cure indicate that achieving gross total resection, immediate postoperative focal radiotherapy, and prolonged combination chemotherapy may be of importance [8]. Effective regimens include cyclophosphamide, topotecan, ifosfamide, doxorubicin, vincristine and dactinomycin. We found that three of the DCS patients in the previous study who had been exposed to high dose rate radiotherapy (≥ 59.9 Gy) had relatively favorable prognosis [8]. In addition, this patient who had been undertaken gross tumor resection, high dose rate radiotherapy, and chemotherapy had no tumor recurrence at the 21-month follow-up. It suggested that the treatment of DCS should include gross total resection and combination of high dose rate radiotherapy (60 Gy) and chemotherapy.

In summary, this first report of DCS with neuronal differentiation extends the immuno-phenotypic spectrum of DCS. Although the prognosis for DCS is poor, gross tumor resection, high dose radiotherapy and chemotherapy may assist in prolonging survival. Long-term observation is essential to better elucidate the biological behavior of DCS with neuronal differentiation.

## Supplementary Information

**Additional file 1: Supplemental Table 1.** The Primary antibodies for immunochemistry and corresponding staining results.

## Data Availability

The data that support the findings of this study are available on request from the corresponding author.

## Abbreviations

DCS: *DICER1*-Associated central nervous system sarcoma
SCS-RMS like-DICER1: Spindle cell sarcoma with rhabdomyosarcoma-like features, DICER1 mutant
WES: Whole-exome sequencing
RNA-seq: RNA sequencing
GO: Gene ontology
MRI: Magnetic resonance imaging
FLAIR: Fluid attenuation inversion recovery
FFPE: Formalin-fixed paraffin-embedded
BWA: Burrows-Wheeler Aligner
TMB: Tumor mutational burden
MSI: Microsatellite instability
VAF: Variant Allele Frequency
LOH: Loss of heterozygosity
