# Supplementary material for: DICER1-associated central nervous system sarcoma with neural lineage differentiation: a case report
Source: Diagn Pathol. 2022 Sep 24;17:72. doi: 10.1186/s13000-022-01252-1 (PMC9508712; doi:10.1186/s13000-022-01252-1)
Supplement: Supplementary file 1 — Additional file 1: Supplemental Table 1. The Primary antibodies for immunochemistry and corresponding staining results. [file 13000_2022_1252_MOESM1_ESM.docx]

**Supplemental Table 1. The Primary antibodies for immunochemistry and corresponding staining results.**

|  | **Primary antibody** | **Clone** | **Brand** | **Dilution** | **Staining Results** |
| --- | --- | --- | --- | --- | --- |
| Commonly diagnostic markers for meningioma, and mesenchymal, non-meningothelial tumours involving the CNS | Desmin | D33 | Dako | 1:50 | + |
|  | MYOD1 | 5.8A | Dako | 1:50 | - |
|  | Smooth muscle actin (SMA) | 1A4 | Dako | 1:400 | - |
|  | Myogenin | F5D | Dako | 1:50 | - |
|  | Vimentin | RV202 | Abcam | 1:150 | + |
|  | TLE1 | sc-9121 | Santa Cruz | 1:100 | - |
|  | STAT6 | E265 | Abcam | 1:500 | - |
|  | CD34 | UMB1 | Abcam | 1:200 | - |
|  | Somatostatin receptor 2A (SSTR2A) | QBEnd10 | Beckman Coulter | 1:500 | - |
|  | Membrane antigen (EMA) | M061301-2 | Dako | 1:50 | - |
| Diagnostic markers for neuroepithelial tumors | Glial fibrill aryacidic protein (GFAP) | 6F2 | Dako Cytomation | 1:500 | - |
|  | S100 | 4c4.9 | Zytomed | 1:3000 | + |
|  | SOX10 | SP267 | Abcam | 1:400 | - |
|  | OLIG2 | H-68 | Santa Cruz | 1:200 | - |
|  | MAP2 | EPR19691 | Abcam | 1:1500 | + |
|  | Synaptophysin (Syn) | EP158 | Bio Genex | 1:100 | + |
|  | Neurofilament protein (NFP) | 2F11 | Dako Cytomatio | 1:150 | + |
|  | Chromogranin A (CgA) | C-12 | Santa Cruz | 1:100 | - |
|  | CD56 | 123C3 | Santa Cruz | 1:200 | + |
|  | Nestin | SP103 | Abcam | 1:100 | + |
|  | SOX2 | EPR3131 | Abcam | 1:100 | + |
| DICER1 and DICER1 sarcoma-associated markers | DICER1 | 4A6 | Abcam | 1:100 | + |
|  | ATRX | HPA001906 | Sigma | 1:800 | + |
|  | H3K27me3 | C36B11 | Cell Signaling Technology | 1:300 | + |
|  | P53 | DO-1 | SantaCruz | 1:200 | - |
|  | Ki-67 | MIB1 | Dako | 1:80 | 70% |
| Other markers | BRG1 | G-7 | Santa Cruz | 1:200 | + |
|  | INI1 | BAF47 | Biosciences | 1:200 | + |
|  | Cytokeratin (CK) | AE1⁄AE3 | Zymed San Francisco | 1:200 | - |
